# Genome-Scale Analysis of Homologous Genes among Subgenomes of Bread Wheat (*Triticum aestivum* L.)

**DOI:** 10.3390/ijms21083015

**Published:** 2020-04-24

**Authors:** Caie Zhou, Zhaonian Dong, Ting Zhang, Jianhui Wu, Shizhou Yu, Qingdong Zeng, Dejun Han, Wei Tong

**Affiliations:** 1 College of Agronomy, Northwest A&F University, Yangling 712100, China; 2 State Key Laboratory of Crop Stress Biology for Arid Areas, Northwest A&F University, Yangling 712100, China

**Keywords:** bread wheat, genome, homologous gene, gene distribution, subgenome

## Abstract

Determining the distribution and correspondence of genome-scale homologous genes in wheat are effective ways to uncover chromosome rearrangement that has occurred during crop evolution and domestication, which can contribute to improvements in crop breeding. High-resolution and comprehensive analysis of the wheat genome by the International Wheat Genome Sequencing Consortium (IWGSC) revealed a total of 88,733 high-confidence homologous genes of four major types (1:1:1, 1:1:0, 0:1:1 and 1:0:1) among the A, B and D subgenomes of wheat. This data was used to compare homologous gene densities among chromosomes, clarify their distribution and correspondence relationship, and compare their functional enrichment. The average density of 1:1:1 homologous genes was about 10 times more than the density of the other three types of homologous genes, although the homologous gene densities of the various chromosomes were similar within each homologous type. Three regions of exceptional density were detected in 1:1:1 homologous genes, the isolate peak on the tail of chromosome 4A, and the desert regions at the start of chromosome 7A and 7D. The correspondence between homologous genes of the wheat subgenomes demonstrated translocation between the tail segments of chromosome 4A and 5A, and the inversion of the segment of original 5A and 7B into the tail of 4A. The homologous genes on the inserting segments of 5A and 7B to 4A were highly enriched in nitrogen, primary metabolite and small molecular metabolism processes, compared with genes on other regions of the original 4A chromosome. This study provides a refined genome-scale reference of homologous genes for wheat molecular research and breeding, which will help to broaden the application of the wheat genome and can be used as a template for research on other polyploid plants.

## 1. Introduction

As an important and widely cultivated crop, bread wheat (*Triticum aestivum* L.) provides about 20% of the total calories in human food [1], and will need to increase its production by about 38% by 2050 to satisfy the increasing food requirements of a growing world population [2]. Hexaploid (AABBDD) wheat contains three similar subgenomes, A, B and D, which come from three ancestral grasses [3], and show a high degree of collinearity and sequence conservation between their homologous genes [4]. The genome of hexaploid wheat (*T. aestivum* L.) is a product of multiple rounds of genome hybridization, for which homologous gene distribution and rearrangement can contribute to understanding the evolution of hexaploid species [5]. Due to a lack of knowledge about genome-scale genetic markers, yield and quality traits, genes and their homologous correspondence between wheat subgenomes, research has been limited to uncovering the underlying molecular mechanisms of crop characteristics, and the effective breeding of fine wheat cultivars to satisfy the increasing requirements of yield and quality.

Wheat genomics research can effectively accelerate the progress of wheat molecular research and breeding. During the initial stages, meiotic pairings were used to reveal the structure of wheat chromosomes [6]. Cellular genetic markers were then developed for wheat homologous gene regions by utilizing full-length wheat cDNA as FISH probes [7]. By comparing the genetic relationship between hexaploid wheat (based on its draft genome sequence) and existing diploid and tetraploid wheat, gene loss after wheat polyploidization was found to be limited, and high structural conservation and sequence similarity between wheat subgenomes was found [8]. Furthermore, the International Wheat Genome Sequencing Consortium (IWGSC) released the chromosome-based reference genome of the wheat cultivar, Chinese Spring, in 2018, which contained 35,345, 35,643 and 34,212 high-confidence genes of the A, B and D subgenomes, respectively. Four types of homologous gene groups (1:1:1, 1:1:0, 0:1:1, and 1:0:1) between the A, B and D subgenomes account for over 85% of the homologous genes, while 1:1:1 homologous genes account for 55% of the homologous genes [9]. There were no significant differences found in gene numbers among the A, B and D subgenomes of wheat, but repeats and gene diversity was lower in the D subgenome [5]. The lower diversity of repeats and genes in the D subgenome may lead to a low chromosome recombination rate and mutation, which may be the main obstacle in effectively utilizing the genetic potential of the D genome in wheat breeding [10]. Comparative analysis of homologous genes between the A, B and D subgenomes of wheat can help us understand wheat chromosome translocation and inversion [11], pericentric rearrangements [12], and the molecular evolution of the α-gliadin gene family, which originated from various subgenomes [13]. Haplotype maps of 890 different wheat terrestrial species were generated using targeted re-sequencing in order to effectively identify genomic regions that contain wild dimer infiltration signals [14]. Similar studies have been done for other proximal species of wheat. The genome arrangements of *Aegilops markgrafii* were detected using cDNA probes [15]. The established linear gene sequence model (genome zipper) indicated that introgressive hybridizations, and/or a series of chromosomal duplications have been important for the speciation and genomic evolution of rye [16]. Hundreds of EST and RFLP markers have been used to show the structural differences between the long arms of barley chromosome 4 and 5 [17]. Comparative analysis of the physical map of *Thinopyrum bessarabicum* with the wheat genome has shown that the 4/5 translocations in *Th. bessarabicum* are also present in the A genome of wheat [18].

The reported distribution and correspondence between homologous genes in wheat were determined based on the wheat draft genome sequence. The order of co-linear genes between wheat and species such as *Brachypodium*, *Triticum urartu* and *Aegilops tauschii*, which have a limited number of homologous genes, and the inferring genes order may not provide an accurate prediction in wheat. Based on the wheat draft shotgun sequence and the genes order between wheat and *Brachypodium*, the comparison of homologous genes is hard to extend to explore structure changes in the whole wheat genome [11], where the 551 homologous genes used to detect pericentric rearrangements in wheat chromosomes [12] are not sufficient for genome-scale investigation. There are still certain questions that have not been fully answered, including whether the genome-scale of homologous genes among the A, B and D subgenomes of wheat are equally distributed on different chromosomes and whether there are differences among the four main types of homologous genes (1:1:1, 1:1:0, 0:1:1, and 1:0:1), regardless of gene distribution density and functional enrichment.

Further investigation of genome-scale distribution and correspondence between homologous genes in wheat can help us to better understand the process of wheat polyploidization to develop more effective genetic markers, isolate genes of important crop traits and provide a springboard for meeting growing food demands [5]. Taking advantage of the high-resolution and comprehensive reference genome and annotation of wheat from IWGSC [9], we compared homologous gene densities among wheat chromosomes, clarified the peak and desert distribution regions of the homologous genes on each chromosome and corresponding functional enrichment. The top four types of high-confidence homologous genes (1:1:1, 1:1:0, 0:1:1 and 1:0:1) in the A, B and D subgenomes of wheat were used to perform analyses, including gene density, distribution, homologous gene correspondence mapping and functional enrichment analyses. This study provides a refined genome-scale reference of homologous genes for molecular research and wheat breeding, which may help to widen the application of the wheat genome and could be used as a template for the analysis of other polyploid plants.

## 2. Results

### 2.1. Homologous Gene Density of Wheat Chromosomes

Based on the number of homologous genes on each wheat chromosome and the chromosome length, the gene number and density of 1:1:1 homologous genes among the A, B and D subgenomes of wheat are presented in Table 1, which shows that the number of 1:1:1 homologous genes in each wheat chromosome group were very similar, and that the gene density of chromosome 5D was the highest (5.4 genes/Mb), while the gene density of chromosome 6B was the lowest (3.0 genes/Mb).

The number and density of 1:1:0 homologous genes in the A, B and D subgenomes of wheat, in which homologous genes were absent on wheat subgenome D, are given in Table 2. For the 1:1:0 type, the homologous gene number on chromosome 3B was the highest at 316, while the gene number in each wheat chromosome group were very similar, except in chromosome group 4 (170 and 209 on 4A and 4B, respectively) and group 5 (314 and 249 on 5A and 5B, respectively). The homologous gene density of chromosome 6A was the highest (0.5 gene/Mb), while that of chromosome 4A was the lowest (0.2 gene/Mb).

Similarly, the number and density of homologous genes with a similarity ratio of 0:1:1 among the A, B and D subgenomes of wheat were calculated, in which homologous genes were absent on wheat subgenome A. The homologous gene number of chromosome 2D was the highest at 435, while the number of chromosome 4B is the lowest at 177. Furthermore, the highest gene density was on chromosome 5D (0.8 gene/Mb) and the lowest was on chromosome 4B (0.3 gene/Mb) (Table 3). 

Finally, a comparison of the gene number and density of 1:0:1 homologous genes, in which homologous genes were absent on wheat subgenome B, found that the lowest gene number was 175 on chromosome 4D, while the highest number was 653 on chromosome 7D. The relative gene density of chromosome 4D was the lowest and that of chromosome 7D was the highest at 0.3 gene/Mb and 1.0 gene/Mb, respectively (Table 4).

### 2.2. Distribution of Homologous Genes on Wheat Chromosomes

#### 2.2.1. Regional Distribution of 1:1:1 Homologous Genes

**Figure 1:**
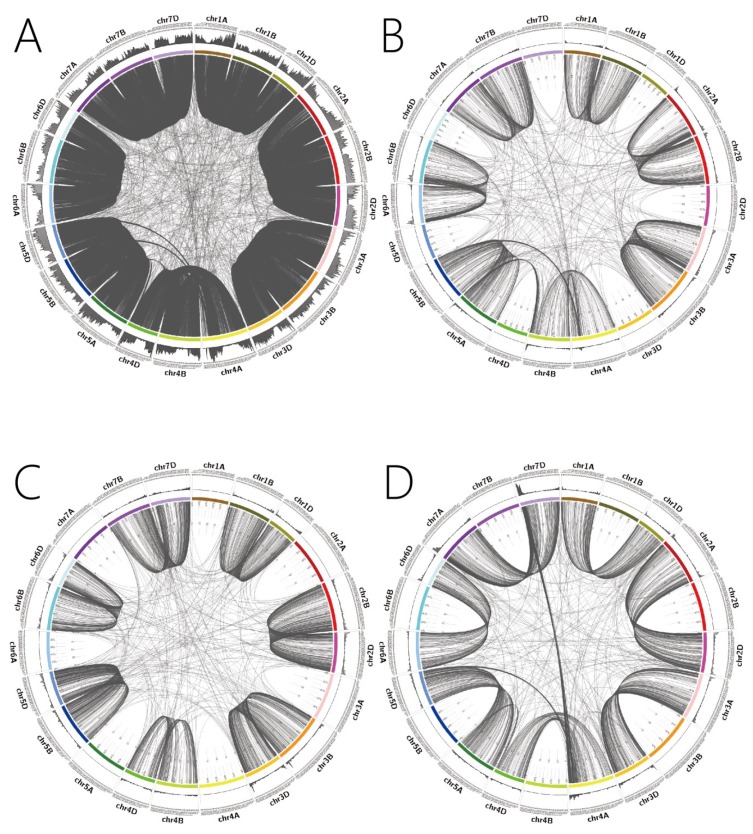
Different types of homologous genes of wheat and their density distribution. The number indicated in the outer circle represents the length of the chromosome. The line chart of the outer circle represents the gene density distribution of each chromosome. Black lines in the inner circle indicate the relationship between homologous genes of the wheat subgenomes. (**A**) Map of 1:1:1 homologous genes between the A, B and D subgenomes of wheat. (**B**) Map of 1:1:0 homologous genes between the A, B and D subgenomes of wheat. (**C**) Map of 0:1:1 homologous genes between the A, B and D subgenomes of wheat. (**D**) Map of 1:0:1 homologous genes between the A, B and D subgenomes of wheat.

The distribution of 1:1:1 homologous genes between A, B and D subgenomes of wheat were plotted and are shown in Figure 1A. The 1:1:1 homologous genes were enriched in region 1–120 M and 300–592 M on 1A, 1–185 M and 340–687 M on 1B and 1–120 M and 303–492 M on 1D; 1–200 M and 460–780 M on 2A, 1–240 M and 400–800 M on 2B and 1–205 M and 400–650 M on 2D; 1–220 M and 455–750 M on 3A, 1–240 M and 420–830M on 3B and 1–190 M and 340–600 M on 3D; 1–200 M and 430–615 M on 4A, 1–200 M and 400–620 M on 4B and 1–150 M and 305–475 M on 4D; 1–140 M and 340–660 M on 5A, 1–150 M and 300–600 M on 5B and 1–140 M and 260–500 M on 5D; 1–250 M and 450–600 M on 6A, 1–290 M and 450–700 M on 6B and 1–280 M and 320–470 M on 6D; 60–300 M and 420–736 M on 7A, 1–250 M and 420–470 M on 7B and 70–290 M and 400–603 M on 7D. The distribution of 1:1:1 homologous genes on the 21 chromosomes showed two enrichment regions on each wheat chromosome, with one being on the head and the other on the tail. The results showed that the homologous gene distribution on wheat chromosome 4A was significantly different to that of other chromosomes.

#### 2.2.2. Regional Distribution of 1:1:0 Homologous Genes

The distribution of 1:1:0 homologous genes among the A, B and D subgenomes of wheat, in which homologues genes were absent in the D subgenome, are shown in Figure 1B, indicating that the 1:1:0 type of homologous genes were enriched in the region of 1–100 M and 500–590 M on chromosome 1A, 1–10 M and 550–687 M on chromosome 1B; 5–100 M and 695–780 M on chromosome 2A, 1–150 M and 635–800 M on chromosome 2B; 5–90 M and 490–750 M on chromosome 3A, 5–90 M and 665–828 M on chromosome 3B; 550–730 M on chromosome 4A, 640–673 M on chromosome 4B; 545–709M on chromosome 5A, 1–230 M and 440–713 M on chromosome 5B; 1–110 M and 550–618 M on chromosome 6A, 5–200 M and 650–720M on chromosome 6B; 1–240 M and 550–736 M on chromosome 7A, 1–210 M and 480–750 M on chromosome 7B. The distribution of 1:1:0 homologous genes on the 14 chromosomes showed two enrichment regions on the ends of the chromosomes, while 4A, 4B and 5A were found to have only one tail enrichment interval. The genes were mainly concentrated on the head, with a few on the tail of chromosome 6A. 

#### 2.2.3. Regional Distribution of 0:1:1 Homologous Genes

The distribution of 0:1:1 homologous genes among the A, B and D subgenomes of wheat, in which the corresponding homologues genes were absent on wheat subgenome A, are shown in Figure 1C, demonstrating that homologous genes were enriched in the region of 5–100 M and 550–689M on chromosome 1B, 5–100 M and 395–495 M on chromosome 1D; 5–200 M and 600–800 M on chromosome 2B, 5–150 M and 550–65 0M on chromosome 2D; 1–100 M and 700–830 M on chromosome 3B, 1–100 M and 500–615M on chromosome 3D; 1–200 M and 500–673 M on chromosome 4B, 1–150 M and 400–509 M on chromosome 4D; 1–100 M and 440–713 M on chromosome 5B, 1–100 M and 330–566 M on chromosome 5D; 1–200M and 600–720 M on chromosome 6B, 5–140 M and 400–473 M on chromosome 6D; 1–200M and 450–750 M on chromosome 7B, 50–200 M and 450–638 M on chromosome 7D. Therefore, it can be seen that the 0:1:1 homologous genes were highly distributed on both ends of the 14 chromosomes.

#### 2.2.4. Regional Distribution of 1:0:1 Homologous Genes

The distribution of 1:0:1 homologous genes among the A, B and D subgenomes of wheat, in which the corresponding homologous genes were absent on wheat subgenome B, are shown in Figure 1D, demonstrating that homologous genes were enriched in the region of 1–100 M and 450–594 M on 1A, 1–70 M and 400–495 M on 1D; 1–200 M and 600–780 M on 2A, 1–140 M and 540–651 M on 2D; 1–100 M and 550–750 M on 3A, 1–100 M and 500–615 M on 3D; 600–744 M on 4A, 1–150 M and 460–509 M on 4D; 1–150 M and 430–709 M on 5A, 1–150M and 330–566M on 5D; 1–200 M and 500–618 M on 6A, 1–150 M and 400–473 M on 6D; 1–200 M and 640–736 M on 7A, 1–100 M on 7D. The two regions with the highest density of 1:0:1 homologous genes were the tail of chromosome 4A (600–744 Mbp) and the start region of chromosome 7D (1–100 Mbp). In addition to chromosome 4A and 7D, the density of homologous genes on 5A (1–150 Mbp and 430–709 Mbp) and 5D (1–150 Mbp and 330–566 Mbp) chromosomes were also high.

### 2.3. Homologous Gene Correlations between the A, B and D Subgenomes of Wheat

The 1:1:1 homologous gene between the A, B and D subgenomes of wheat showed a high degree of correspondence in the same chromosome group, while a small section of 4A genes showed obvious differences, which were highly homologous with 5B. In addition, a small number of 4D genes also showed obvious differences, which were highly homologous with 5A (Figure 1A). 

Similarly, the gene correspondence map of homologous genes with a similarity ratio of 1:1:0 among the A, B and D subgenomes of wheat was obtained (Figure 1B). The correspondence map of 1:1:0 homologous genes showed that the degree of correspondence among genes on 14 chromosomes was very high, while 4A, 4B, 5A and 5B showed significant differences. A small number of 4A genes showed an obvious difference, which highly corresponded with that of 5B. Significant differences were also found for a small number of 4B genes, which highly corresponded with that of 5A.

Additionally, the correspondence map of 0:1:1 homologous genes in the A, B and D subgenomes of wheat (Figure 1C) showed that the homologous genes had a high degree of correspondence. There were some genes that corresponded with that of 7B on chromosome 5D, with significant differences. 

Finally, the 1:0:1 homologous gene correspondence map in the A, B and D subgenomes of wheat showed that most homologous genes were represented in the same chromosome group without the corresponding genes in the B subgenome (Figure 1D). The only significant exception was the correspondence between homologous genes on the tail of chromosome 4A and that of 5D and the start of 7D. 

**Figure 2:**
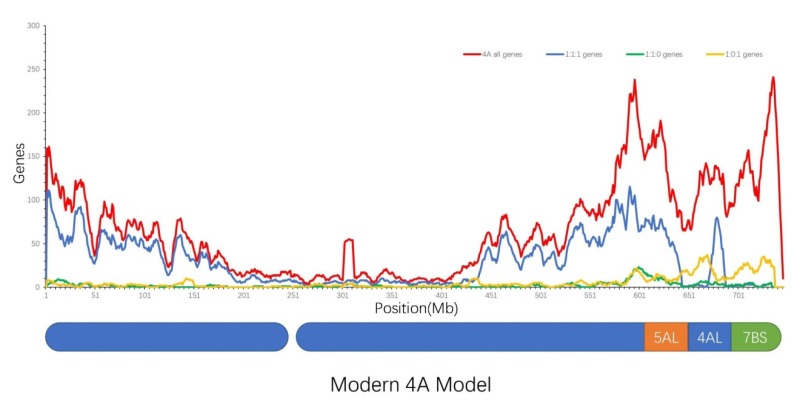
Density distribution of the different types of homologous genes on wheat chromosome 4A. The density distribution of the different types of homologous genes on wheat chromosome 4A are presented in Figure 2. The blue rounded rectangles represent the short and long arms of wheat chromosome 4A, the white space represents the centromere, the orange segment represents the insertion from the original wheat chromosome 5AL, while the green segment represents the insertion from the original wheat chromosome 7BS. The blue curve represents the density distribution of 1:1:1 homologous genes, the green curve represents the distribution of 1:1:0 genes, the yellow curve represents the distribution of 1:0:1 genes, and the red curve represents the density distribution of all the genes on chromosome 4A.

We extended the original segments on wheat chromosome 4A, as reported by Jian Ma [12], using over 2000 homologous genes (1:1:1) of the chromosome, and then mapped the distribution of 1:1:1 homologous genes on the segmental chromosome 4A to determine why there was a significant gene enrichment peak on the tail of 4A, which was the only region with a large-scale chromosome transition among the A, B and D subgenomes of wheat. The blue curve in Figure 2 represents the distribution of 1:1:1 genes on chromosome 4A, which was significantly in accordance with that of the original segments of chromosome 4A. The isolated peak of the 1:1:1 homologous genes on the tail of chromosome 4A was only located in the original 4AL segment, and the nearby gene desert regions belonged to the original 5AL and 7BS insertion segments, while the gene distribution of the other three types of homologous genes, 1:1:0, 0:1:1 and 1:0:1, did not show a similar peak curve on the same tail region of chromosome 4A (Figure 2). However, the complete genes distribution of 4A did not display a similarly isolated peak curve, as indicated by the red curve shown in Figure 2.

### 2.4. Gene Ontology Analysis of Wheat Homologous Genes

**Figure 3:**
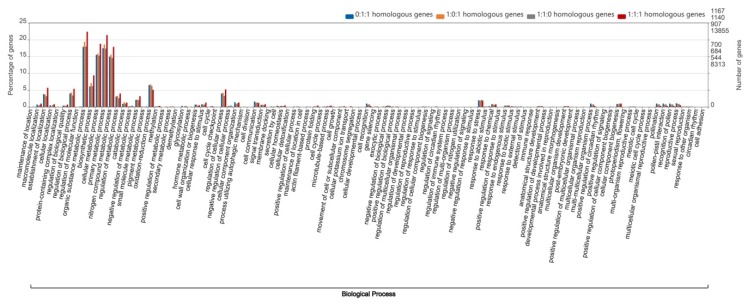
Gene ontology (GO) comparison analysis of the four types of homologous genes between the wheat subgenomes. The vertical line on the left represents the percentage of genes. The ordinate on the right represents the number of genes. The blue, orange, gray and red bars represent the GO catalogs of homologous genes with a similarity ratio of 0:1:1, 1:0:1, 1:1:0 and 1:1:1, respectively, in the A, B and D subgenomes of wheat.

Analysis of the chromosome location of homologous genes can provide information on differences in their distribution on various wheat chromosomes. Furthermore, the functional analysis of different ratios of homologous genes between the A, B and D subgenomes of wheat (ratios of 1:1:1, 1:0:1, 1:1:0 and 0:1:1) can be used to explore whether their functions are different. In order to obtain a map of the gene function enrichment analysis, wego software (http://wego.genomics.org.cn/) along with the gene ontology (GO) IDs corresponding to the gene ID were used to obtain the functional enrichment map of 1:1:1 homologous genes. The top two biological processes were identified as the metabolism process and the cellular process. Similarly, the GO enrichment map of 1:1:0 homologous genes, the GO enrichment map of 0:1:1 homologous genes, and the GO biological process catalog of 1:0:1 homologous genes all showed that the top two functions that were enriched were the metabolism process and cellular process (Figure 3).

**Figure 4:**
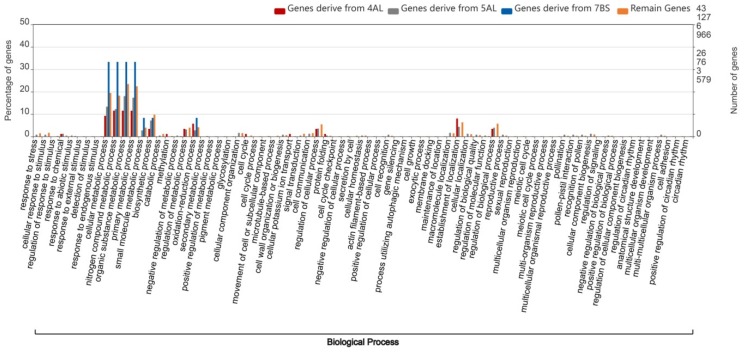
GO comparison analysis of 1:1:1 homologous genes on different segments of wheat chromosome 4A. Figure 4 presents the GO functional comparison map of 1:1:1 homologous genes among wheat subgenomes on four segments of wheat chromosome 4A, the original 4AL segment (red bar), the original 5AL segment (grey bar), the original 7BS segment (blue bar), and the remaining segment (orange bar). The vertical line on the left represents the percentage of genes. The ordinate on the right represents the number of genes, while the numbers from top to bottom correspond with that of gene groups from left to right.

**Figure 5:**
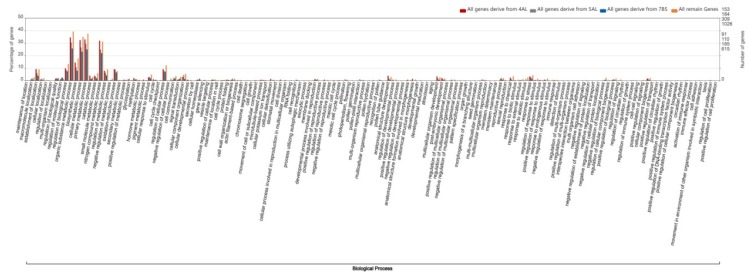
GO comparison analysis of all genes on different segments of wheat chromosome 4A. Figure 5 shows the GO functional comparison map of all the genes on the four segments of wheat chromosome 4A, the original 4AL segment (red bar), the original 5AL segment (grey bar), the original 7BS segment (blue bar), and the remaining segment (orange bar). The vertical line on the left represents the percentage of genes. The ordinate on the right represents the number of genes, while the numbers from top to bottom correspond with that of gene groups from left to right.

In addition to the global GO functional catalog analysis of the four types of homologous genes of wheat, we also explored the genes’ functional enrichment in the various original segments of the modern wheat chromosome 4A, which displayed the most significant transition and insertion among chromosomes 4, 5 and 7. Based on the 1:1:1 gene distribution peak on the tail of chromosome 4A and the original insertion of segments of 5AL and 7BS into the 4A tail, as indicated by the order of corresponding homologous genes (Supplementary Materials, Table S1), we divided the 1:1:1 homologous genes of chromosome 4A into four segments, original 4AL, 5AL, 7BS and the remaining segment, and then compared them with their GO catalogs. For the 1:1:1 homologous genes on the four chromosome 4A segments, the homologous genes on the inserted 5AL and 7BS segments were enriched in nitrogen compound metabolic process, organic substance metabolic process, primary metabolic process and small molecular metabolic process, and were more significantly enriched than the homologous gene on the original 4AL segment. With regard to the biological processes of the four GO catalogs, the homologous genes on the inserted 7BS segment were more significantly enriched than those of the remaining segment (Figure 4). However, in the GO catalog analysis for all genes of the four segments of chromosome 4A, the functional enrichment differences in the above four metabolic catalogs disappeared, and genes on the original 4AL segment were found to be even more highly enriched than genes on the original 5AL and 7BS segments (Figure 5).

## 3. Discussion

The four types of homologous genes (1:1:1, 1:1:0, 0:1:1, and 1:0:1) found in the A, B and D subgenomes of wheat accounted for over 85% of all homologous gene groups [9], and our research further investigated the density of homologous genes among wheat chromosomes, their regional distribution variation on all wheat chromosomes, and the differences in functional enrichment. The average chromosome density of 1:1:1 type of homologous genes was 4.0 genes/Mb, which was almost 10 times higher than the average density of the other three types of homologous genes, which showed densities of 0.35, 0.53 and 0.51 genes/Mb for the 1:1:0, 0:1:1, 1:0:1 types of homologous genes, respectively. Three copies of the main homologous genes were found on the A, B and D subgenomes of wheat, and there were about 10 times more of these compared to other homologous genes that had lost a copy on certain subgenomes, irrespective of whether it was the 1:1:0, 0:1:1, or 1:0:1 type. For most chromosomes, the gene densities of all four types of homologous genes were very similar, although some differences were found such as the 1:1:1 homologous genes density of chromosome 5D (5.4 genes/Mb), which was 1.8 times higher than the density of 6B (3.0 genes/Mb) (Table 1); the density of 1:1:0 homologous genes of chromosome 6A (0.5 genes/Mb), which was 2.5 times higher than the density of 4A (0.2 genes/Mb) (Table 2); the density of 0:1:1 homologous genes of chromosome 5D (0.8 genes/Mb), which was 2.7 times higher than the density of 4B (0.3 genes/Mb) (Table 3); and the density of 1:0:1 homologous genes of chromosome 7D (1.0 genes/Mb), which was 3.3 times higher than the density of 4D (0.3 genes/Mb) (Table 4). 

Except for certain original genes-rich chromosomes, such as the chromosome 5 group, our results did not identify specific wheat chromosomes with an enrichment of homologous genes. Then, we examined whether homologous gene enrichment or desert regions could be found on various wheat chromosomes. For a majority of the 1:1:1 type homologous genes, the distribution curves on all wheat chromosomes are similar and the homologous gene density of the centromere region was at the bottom of the distribution curve, with an increase in density towards both ends. However, as shown by the density distribution, there were three exceptionally large regions of genes: the tail of chromosome 4A, which displayed an isolated peak at a gene density of 650–750 Mb, and the start region of chromosome 7A (0–75 Mb) and 7D (0–60 Mb), which both displayed a desert region of homologous genes, which is shown in the outer circle of Figure 1A. The density distribution curves of the other three types of homologous genes on wheat chromosomes also show a similar curve (the centromere regions have the lowest density with an increase towards both ends). However, the distribution of 1:0:1 homologous genes on the tail of chromosome 4A showed a double peak, and the distribution of 1:0:1 genes at the start of chromosome 7D showed an extremely high density (outer circle in Figure 1D).

In addition to the distribution of homologous genes, we were eager to clarify whether the three unusual distribution regions of 1:1:1 homologous genes on the tail of chromosome 4A and the start of 7A and 7D, were a result of random or inherent phenomena. We also wanted to determine whether there was a correlation between the three exceptional regions of densely distributed 1:1:1 genes and the unusual distribution mode of 1:0:1 genes on the same tail of chromosome 4A and the start of 7D. We drew correlation circus maps of all homologous genes of the wheat chromosomes, and integrated them with gene density maps for all four types of homologous genes (Figure 1), in which most homologous genes displayed highly dense correlation lines between the same chromosome group. However, there were two exceptional regions for 1:1:1 genes, one was the tail of chromosome 4A, which was found to be correlated with homologous genes located on the tail of 5B, 5D and the start of 7D, and the other was the tail of 5A, which was found to be correlated with homologous genes located on the tail of 4B and 4D (Figure 1A). For the 1:1:0 homologous genes, there were two exceptional regions, the tail of chromosome 4A, which was found to be correlated with the tail of 5B, and the tail of 5A, which was found to be correlated with the tail of 4B (Figure 1B). For the 1:0:1 homologous genes, the same tail region of chromosome 4A and 5A showed exceptional correlation, while homologous genes on the tail of 4A were found to be correlated with the tail of 5D and the start of 7D, and homologous genes on the tail of 5A were found to be correlated with the tail of 4D (Figure 1D). Most of the 0:1:1 homologous genes showed a high degree of correlation between the same wheat chromosome group, without significant exceptions (Figure 1C). In summary, the genome-scale correlation map of homologous genes revealed that there were three significant correlations between different chromosome groups in the wheat genotypes of Chinese Spring. The first was the homologous gene correlation between the tail of 4A and the tail of 5B and 5D (1:1:1, 1:1:0 and 1:0:1), the second was the homologous gene correlation between the tail of 5A and the tail of 4B and 4D (1:1:1, 1:1:0 and 1:0:1), while the third was the homologous gene correlation between the tail of 4A and the start of 7D (1:0:1). 

These three correlated regions among genome-scale homologous genes indicated that there were two transformations or insertions that had occurred between different wheat chromosome groups: (1) The homologous gene correlation between the tail of 4A and the tail of 5B and 5D, and the gene correlation between the tail of 5A and the tail of 4B and 4D proved that a large transformation had taken place between the tail of chromosome 4A and 5A (603- 641MB in chromosome 4A). The transformation between the tail of chromosome 4A and 5A clearly explains the presence of a large interlaced correlation of homologous genes between the tail of 4A and that of 5B and 5D, with a similar interlaced correlation between the tail of 5A and that of 4B and 4D, which in fact are the only two significantly crossed correlations among 1:1:1 homologous genes of the different wheat chromosome groups. This large-scale transformation also provides a good explanation for the presence of an interlaced correlation between the tail of 4A and 5B, and a crossed correlation between the tail of 5A and 4B (Figure 1B), as well as an interlaced correlation between the tail of 4A with 5D, and a crossed correlation between the tail of 5A and 4D (Figure 1D). (2) Homologous gene correlation between the tail of chromosome 4A and the start of 7D indicate the presence of an insertion of the original chromosome 7B segment into the tail of 4A, which is consistent with the report by Jian Ma [12]. On the chromosome arm 4AL, a paracentric inversion was detected [19,20]. Using the deletion stocks of wheat, the spreading of RFLPs markers along the wheat group 4 chromosomes was used to identify rearranged segments on chromosome 4A [21,22]. By comparing the genome sequences of wild emmer wheat and *A. tauschii*, a novel scenario of the evolution of rearranged wheat chromosomes 4A, 5A and 7B was identified [23].

This large insertion of an original 7B segment also explains the presence of the only significantly crossed correlations among 1:0:1 homologous genes, between the tail of 4A (which was in fact an inserted segment of the original wheat 7B segment) and the start of 7D, whose homologous genes were correlated with genes on the start of chromosome 7A (Figure 1D). The insertion of the original 7B segment into the tail of 4A also provides a good explanation for the presence of three regions with exceptional density among 1:1:1 homologous genes. As shown in Figure 2, the isolated peak of homologous genes on the tail of 4A is an exact copy of the original 4AL segment, whose genes were confirmed to be correlated with genes on 4B and 4D, and the homologous genes on the end of the modern 4A segment (the original 7BS insertion segment) were correlated with genes on the start of 7A and 7D. Thus, homologous gene correlation between the 4A tail (original 7B segment), 7A start and 7D start were confirmed to not belong to 1:1:1 genes between A, B and D subgenomes, which may explain the desert region of 1:1:1 genes distributed on the end of 4A. The 1:1:1 genes desert regions on the start of 7A (0–75 Mb) and 7D (0–60 Mb) have few homologous genes because the original 7B segment containing corresponding homologous genes was cut and inserted into the end of 4A. In fact, genes on the three desert regions of 1:1:1 homologous genes (the end of 4A, the start of 7A and 7D), are essentially highly correlated, as shown through the 1:0:1 genes correlation between the end of 4A and the start of 7D, as well as the correlation between the start of 7D and start of 7A (Figure 1D). Thus, the nominal correlations between the end of 4A, the starts of 7A and 7D, were found to not belong to 1:1:1 genes in the A, B and D subgenomes, which may provide a good explanation for the presence of three significant desert regions of 1:1:1 genes.

Meiotic recombination is an effective and quick way to increase offspring variability and adaptation, which, in fact, is not a random event and crossovers are formed in the distal half of chromosomes [24]. Therefore, the insertion of 5AL and 7BS segments into the tail of chromosome 4AL can help to increase its crossover frequency. The sequence analysis of wheat chromosome 3B also indicated that wheat-specific inter-chromosomal and intra-chromosomal gene duplication activities may be potential sources of variability for adaption [25].

In addition to their relationship with the distal half of chromosomes, crossover frequencies have been reported to be specific to chromosome segments and independent of the location of the segment, based on wheat chromosome arms 2BS and 4AL, which were inverted in reverse tandem duplications [26]. The GO functions of the four types of homologous genes were similarly enriched in organic substance, cellular metabolic, primary metabolic and nitrogen compound metabolic processes, with the GO enrichment of 1:1:1 genes being slightly higher than the other three types of homologous genes (Figure 3). Furthermore, we explored the specificity of the insertion of segments of 5AL and 7BS into 4A. The GO enrichment results showed that the homologous genes on the inserting 5AL and 7BS segments were significantly more highly enriched in nitrogen compound, organic substance, small molecular and primary metabolic processes, than homologous genes of the other regions of chromosome 4A. The percentage of homologous genes in the original segment of 4AL in the above four GO catalogs were the lowest, but the percentage of homologous genes in the GO catalogs (regulation of metabolic process, oxidation-reduction process, cell cycle, protein folding and establishment of localization) was higher than in the other 4A segment regions (Figure 4). Carbon and nitrogen metabolism are the most fundamental metabolic processes in plants [27]. Nitrogen metabolism has been found to be closely related to crop yield and important agronomic quality indicators [28,29,30], while the metabolism of organic substances has been identified during wheat anther development [31]. 

The concentration of the original segments of 5AL and 7BS with highly enriched metabolism genes on the narrow end region of chromosome 4AL can also help the offspring of wheat (Chinese Spring) to retain their metabolic function and increase their crossover frequency with other proximal regions through the meiotic recombination process. Conversely, many agronomic genes are located in chromosome crossover-poor regions and chromosome structural rearrangements can help to increase the recombination frequency in crossover-poor regions and develop strategies for the introgression of useful genes into crops [24]. The process used for this genome-scale homologous genes analysis provides an effective way to decipher the correspondence of homologous genes and chromosome rearrangement that occurs during crop evolution or the domestication process, especially for polyploid *Triticum* species, which can contribute to the field of molecular crop breeding.

## 4. Materials and Methods 

### 4.1. Experimental Materials

Analysis of the wheat genome by IWGSC revealed the distribution of key elements and a detailed comparison of homologous genes between the A, B and D subgenomes (https://urgi.versailles.inra.fr/download/iwgsc/IWGSC_RefSeq_Annotations/v1.0/). Based on the wheat genome annotation v1.0 [9], a total of 181,036 genes (103,757 HC genes and 77,279 LC genes) were included. Since both HC genes and LC genes were included in the analysis, the resulting homologous genomes were classified into three types: “HC only”, “LC only”, and “mixed” [9]. This study utilized the supplementary materials and the high-confidence gene annotation files of the wheat genome [9]. In addition, the annotation file for the wheat homologue group, which contained homologous gene information on LC-only, HC-only, and mixed multiple similarity ratios among the three subgenomes of hexaploid wheat, was also obtained from IWGSC [32]. We used Excel to screen for information that met the conditions: “HC-only & 1:1:1”, “HC-only & 1:0:1”, “HC-only & 1:1:0”, “HC-only & 1:1:0” and “HC-only & 0:1:1”, from the annotation file of the wheat homologous group to obtain information on homologous genes with the ratio of 1:1:1, 1:1:0, 1:0:1 and 0:1:1 between the A, B and D subgenomes. 

### 4.2. Parameter Optimization for Illustrating Gene Distribution

The parameter optimization process used to illustrate the gene distribution of 1:1:1 homologous genes on chromosome 1A is given as an example. The distribution of homologous genes on each chromosome was obtained by changing the size of the window and the sliding step using R3.5.2. Six pairs of windows and sliding step-size parameters were evaluated (1000 K window with 500 k step, 1000 K window with 900 k step, 2 M window with 1 M step, 5 M window with 1 M step, 10 M window with 1 M step and 10 M window with 5 M step). Line smoothness, discrimination and visualization were used to evaluate the parameters, and the standard parameters were determined to be a window of 10 M and a sliding step of 1M (Supplementary Materials, Figure S1, Distribution density map of different window and sliding step sizes). 

### 4.3. The Distribution and Correspondence (Circle Map) between Homologous Genes

The distribution and correspondence map of homologous genes was created using Circos software v0.69-9 (http://circos.ca/software/download/) and strawberry Perl v5.16 software (http://strawberryperl.com/), and were based on gene density calculations (calculated by the homologous gene number from the wheat annotation v1.0 and the confirmed window sizes in 4.2), chromosome lengths and homologous gene relationships.

### 4.4. GO Analysis

Based on the corresponding GO ID file of wheat genes obtained from Ensembl Plants (BioMart), we extracted the GO IDs of homologous genes using a script developed by us (Supplementary Materials, software File S1). Then, wego software (http://wego.genomics.org.cn/) was utilized to perform GO comparison analyses and create the GO bar charts.

## Figures and Tables

**Table 1:** Distribution of 1:1:1 homologous genes among wheat A, B and D subgenomes.

Chromosome Name	Total Number of Homologous Genes	Chromosome Length (Mb)	Homologous Gene Density (Gene Number/Mb)
1A	2451	594.10	4.1
1B	2455	689.85	3.6
1D	2453	495.45	5.0
2A	3186	780.80	4.1
2B	3183	801.26	4.0
2D	3186	651.85	4.9
3A	2911	750.84	3.9
3B	2928	830.83	3.5
3D	2918	615.55	4.7
4A	2287	744.59	3.1
4B	2295	673.62	3.4
4D	2301	509.86	4.5
5A	3081	709.77	4.3
5B	3058	713.15	4.3
5D	3060	566.08	5.4
6A	2140	618.08	3.5
6B	2156	720.99	3.0
6D	2143	473.59	4.5
7A	2418	736.71	3.3
7B	2399	750.62	3.2
7D	2413	638.69	3.8

**Table 2:** Distribution of 1:1:0 homologous genes among wheat A, B and D subgenomes.

Chromosome Name	Total Number of Homologous Genes	Chromosome Length(Mb)	Homologous Gene Density (Gene Number/Mb)
1A	200	594.10	0.3
1B	208	689.85	0.3
2A	294	780.80	0.4
2B	292	801.26	0.4
3A	293	750.84	0.4
3B	316	830.83	0.4
4A	170	744.59	0.2
4B	209	673.62	0.3
5A	314	709.77	0.4
5B	249	713.15	0.3
6A	297	618.08	0.5
6B	305	720.99	0.4
7A	246	736.71	0.3
7B	235	750.62	0.3

**Table 3:** Distribution of 0:1:1 homologous genes among wheat A, B and D subgenomes.

Chromosome Name	Total Number of Homologous Genes	Chromosome Length (Mb)	Homologous Gene Density (Gene Number/Mb)
1B	291	689.85	0.4
1D	298	495.45	0.6
2B	425	801.26	0.5
2D	435	651.85	0.7
3B	416	830.83	0.5
3D	408	615.55	0.7
4B	177	673.62	0.3
4D	183	509.86	0.4
5B	405	713.15	0.6
5D	426	566.08	0.8
6B	301	720.99	0.4
6D	293	473.59	0.6
7B	320	750.62	0.4
7D	292	638.69	0.5

**Table 4:** Distribution of 1:0:1 homologous genes among wheat A, B and D subgenomes.

Chromosome Name	Total Number of Homologous Genes	Chromosome Length (Mb)	Homologous Gene Density (Gene Number/Mb)
1A	218	594.10	0.4
1D	230	495.45	0.5
2A	366	780.80	0.5
2D	371	651.85	0.6
3A	322	750.84	0.4
3D	330	615.55	0.5
4A	400	744.59	0.5
4D	175	509.86	0.3
5A	272	709.77	0.4
5D	284	566.08	0.5
6A	236	618.08	0.4
6D	238	473.59	0.5
7A	467	736.71	0.6
7D	653	638.69	1.0

## Supplementary Materials

Supplementary materials can be found at https://www.mdpi.com/1422-0067/21/8/3015/s1.
