# Supplementary material for: Genome-Scale Analysis of Homologous Genes among Subgenomes of Bread Wheat (Triticum aestivum L.)
Source: Int J Mol Sci. 2020 Apr 24;21(8):3015. doi: 10.3390/ijms21083015 (PMC7215433; doi:10.3390/ijms21083015)
Supplement: Supplementary file 1 [file ijms-21-03015-s001.zip › Supplements/Supplemental Figure legend.docx]

**Figure S1. The homologous genes density distribution of wheat chromosome 1A under various parameters.**

The 1:1:1 homologous genes on the wheat chromosome 1A were selected as example to determine the proper window parameters. We tested 6 pairs of window parameters for the homologous genes density map by using R package: (a)window of 1000 kb, moving step of 500 kb; (b)window of 1000 kb, moving step of 900 kb; (c)window of 2 Mb, moving step of 1 Mb; (d)window of 5 Mb, moving step of 1 Mb; (e)window of 10M, moving step of 1 Mb; (f)window of 10 Mb, moving step of 5 Mb. According to the comprehensive evaluation of line smoothness, discrimination and visualization, the parameter standard is determined as window 10M and moving step of 1 Mb.
