# Supplementary figures and images for: Genome-Scale Analysis of Homologous Genes among Subgenomes of Bread Wheat (Triticum aestivum L.)
Source: Int J Mol Sci. 2020 Apr 24;21(8):3015. doi: 10.3390/ijms21083015 (PMC7215433; doi:10.3390/ijms21083015)

(a)

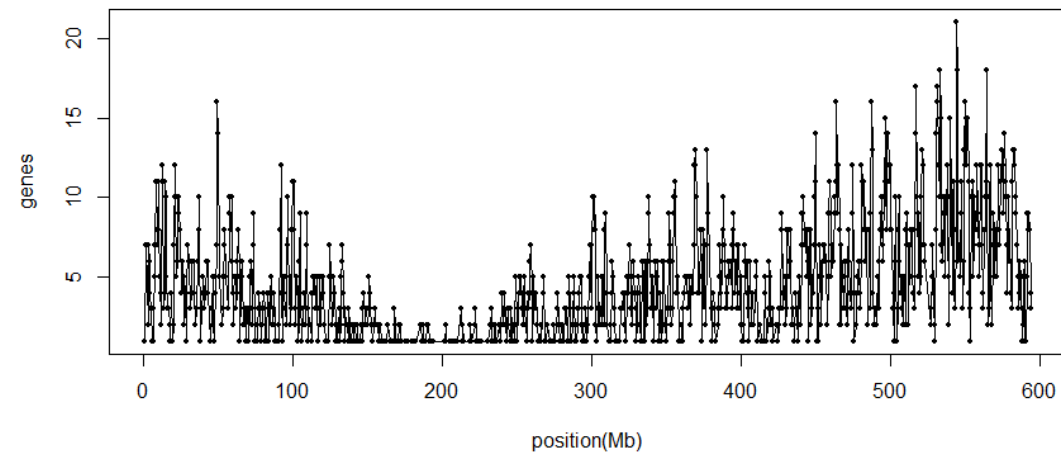

(b)

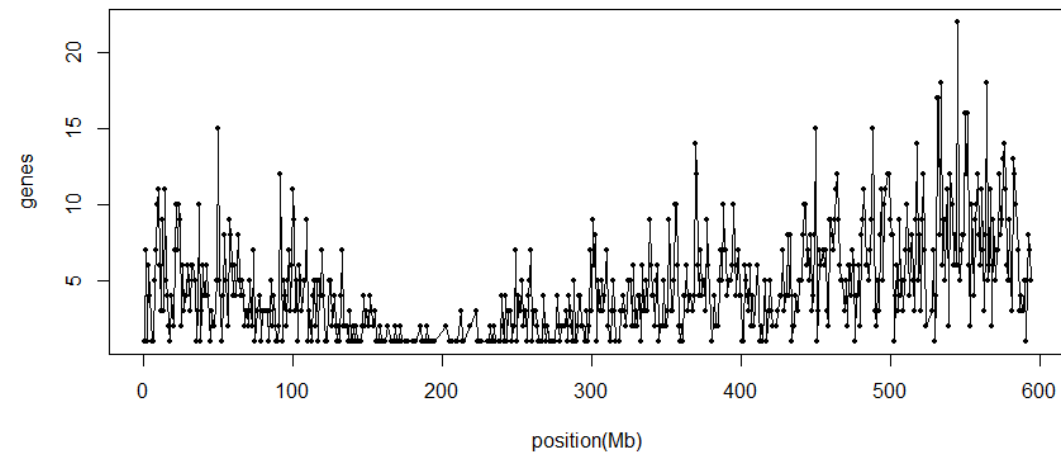

(c)

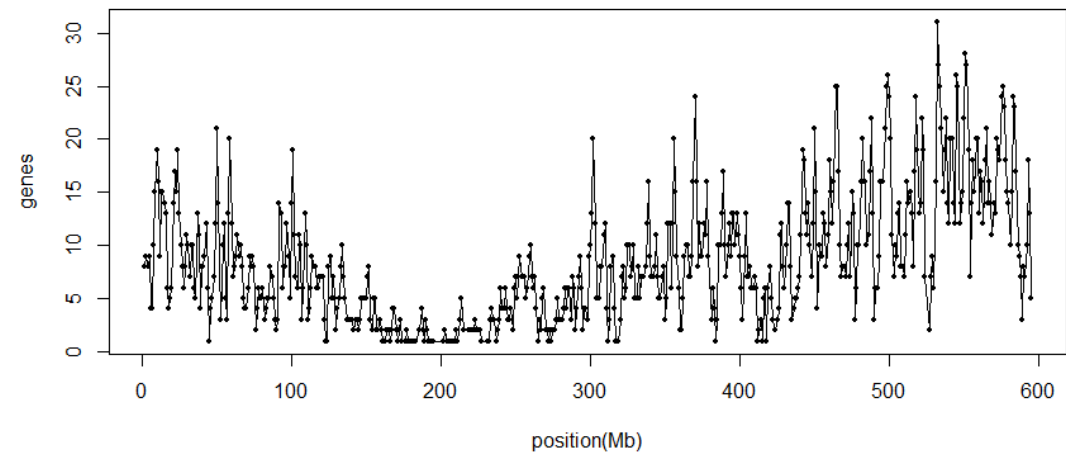

(d)

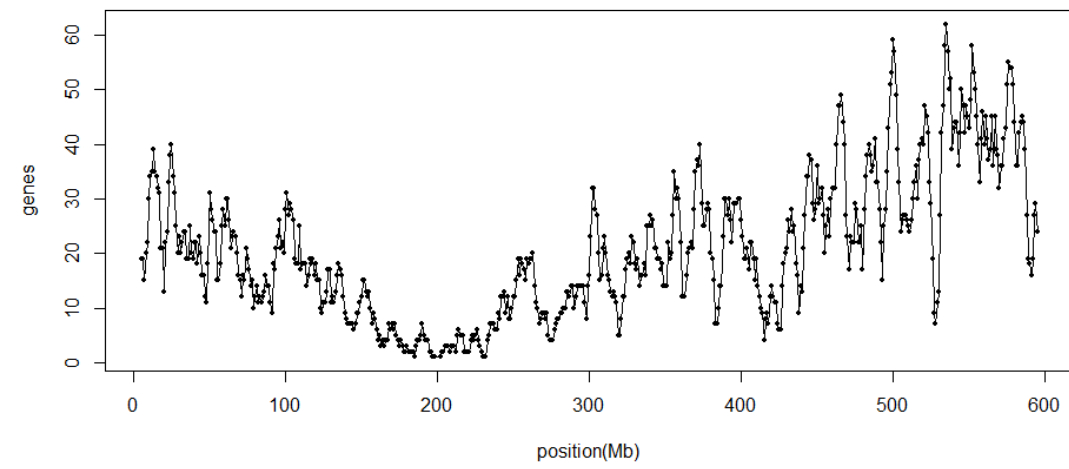

(e)

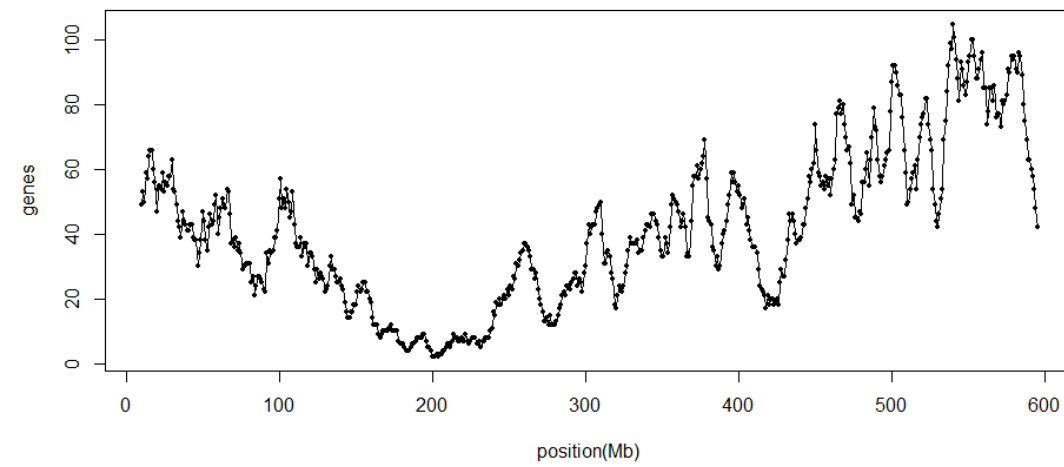

(f)

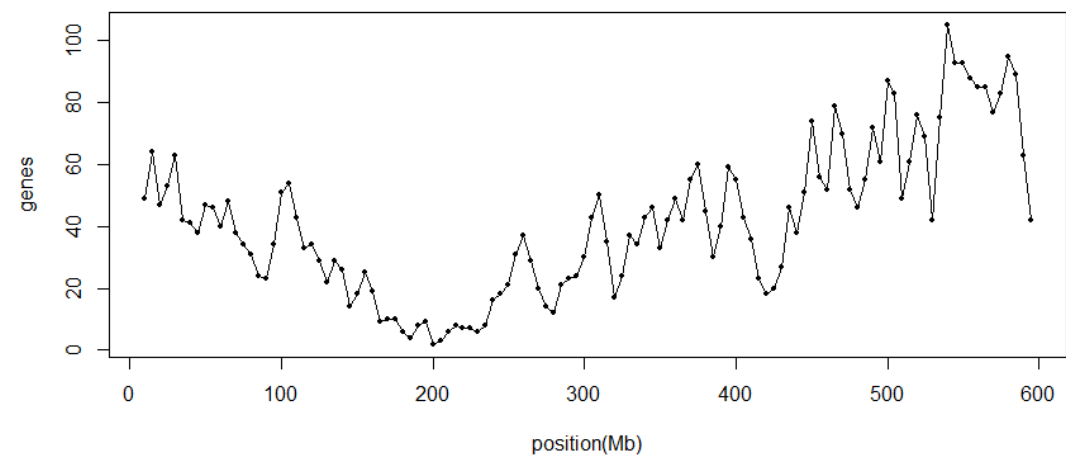

Supplement: Supplementary file 1 [file ijms-21-03015-s001.zip › Supplements/Supplemental FigureS1.pdf]
